# Three-dimensional substructure measurements for the differential diagnosis of ground glass nodules

**DOI:** 10.1186/s12890-017-0438-y

**Published:** 2017-06-19

**Authors:** Mingzheng Peng, Gang Yu, Chengzhong Zhang, Cuidi Li, Jinwu Wang

**Affiliations:** 1 Shanghai Key Laboratory of Orthopaedic Implant, Department of Orthopaedic Surgery, Shanghai Ninth People’s Hospital, Shanghai Jiao Tong University School Of Medicine, Room 703, No. 3 Building, 693 Zhizaoju Road, Shanghai, 200011 China; 2 Department of Anesthesiology, Binzhou Central Hospital, Binzhou Medical College, Binzhou, China; 3 Department of Radiology, Shanghai First People’s Hospital, Shanghai Jiao Tong University School of Medicine, Shanghai, China; 4 School of Biomedical Engineering, MED-X Research Institute of Shanghai Jiao Tong University, Shanghai, China

**Keywords:** Peak, Substructure, Ground-glass nodule, Follow-up, Three-dimensional, Lung cancer

## Abstract

**Background:**

We analyzed the differences between maximum and peak computed tomography (CT) numbers (M-P), respectively representing the densities of the solid center and the main periphery of ground-glass nodules (GGNs), and the average change in M-P velocity (V(M-P)) during follow-up to differentiate between pre-invasive (PIA) and invasive adenocarcinoma (IAC).

**Methods:**

Data of 102 patients were retrospectively collected and analyzed in our study including 43 PIAs and 59 IACs. Diameters, total volumes, and the maximum and peak CT numbers in CT number histograms were measured and followed for at least 3 months. This study was registered retrospectively.

**Results:**

The M-P values for IACs were higher than those for PIAs (*p* = 0.001), with an area under the curve (AUC) of 0.810 and a threshold of 489.5 Hounsfield units (HU) in ROC analysis. The V(M-P) values for IACs were smaller than those for PIAs (*p* = 0.04), with an AUC of 0.805 and a threshold of 11.01 HU/day.

**Conclusions:**

M-P and V(M-P) values may help distinguish IACs from PIAs by representing the changes in the sub-structural densities of GGNs during follow-up.

## Background

Lung carcinoma is well-known to be one of the most malignant cancers due to its remarkable morbidity and mortality [1]. Owning to the current prevalence of low-dose computed tomography (CT) scans, ground-glass nodules (GGNs) are regularly encountered in individuals during physical examinations or general lung cancer surveys. Thus, the ability to provide a differential diagnosis of GGNs, regarded as precursors of lung carcinoma, is both imperative and meaningful.

Periodic follow-up CT scans play a crucial role in the diagnostic evaluation of GGNs. Some authors have assessed GGN volume-doubling times (VDTs) as part of their pathologic evaluations. However, internal growth features are not considered as part of the VDT, especially for sub-solid nodules. Additionally, GGN volumes may be affected by lung volumes and some lesions fail to demonstrate progression in diameter or volume over several years [2]. The progression of pulmonary adenocarcinoma, manifesting as sub-solid nodules, is reflected by increases in lesion size and internal growth that result in increased CT attenuation due to increases in the percentage of solid components [3].

In the present study, we measured the peak CT number (PEAK) in a histogram of CT numbers distributed throughout the GGN and the maximum CT attenuation number (MAX) in nodules during follow-up. The PEAK value represents the highest CT number associated with pixels in a transverse section of the GGN histogram whereas the MAX represents the highest longitudinal CT number in the nodule histogram. We then defined two additional parameters: M-P, which equals the MAX minus PEAK value, and V(M-P), which reflects the average velocity of change of the M-P parameter during follow-up. These parameters were tested for their ability to distinguish between pre-invasive adenocarcinomas (PIAs) and invasive adenocarcinomas (IACs).

## Methods

### Patients

We retrospectively collected clinical, radiological, and pathological data for 102 patients treated at our hospital between 2012 and 2015. All of the patients had been informed that one or more GGNs (<3 cm in diameter) were detected in their CT screening results; none of the patients had previous primary or metastatic lung carcinoma. All patients received a two-week course of antibiotics, and were recommended to undergo a follow-up CT scan three or more months later. The follow-up periods differed among patients, but were ≥3 months; our data were collected from the last two CT scans prior to surgery and were also separated by 3 months. With regard to short-term follow-up, the British Thoracic Society and Fleischner Society guidelines suggest an initial follow-up CT scan 3 months after detection [4]. Only patients with stable or growing lesions, after antimicrobial treatment, were included in the study; those with transient lesions that were successfully treated with the antimicrobial (those with inflammatory lesions) were excluded [5]. The exclusion criteria were as follows: patients with (1) lesions having average diameters >3 cm, along three axes; (2) small cell lung carcinoma, squamous carcinoma, or metastatic carcinoma; (3) a preoperative follow-up interval of <3 months.

### Pathological examination

Surgically resected GGN specimens were routinely fixed in 10% formalin and processed in paraffin blocks for pathologic examination. Tissue sections (4-μm thick), including the largest cut tumor surface, were stained with hematoxylin and eosin. According to the new International Association for the Study of Lung Cancer/American Thoracic Society/European Respiratory Society classification of pulmonary adenocarcinoma, all nodules were classified as PIAs (including atypical adenomatous hyperplasia [AAH] and adenocarcinoma in situ [AIS]) or IACs (including minimally invasive adenocarcinoma [MIA]).

### CT Imaging

CT images were acquired using various instruments, including a Somatom Plus 4 (Siemens, Erlangen, Germany), LightSpeed Ultra (GE Medical Systems, Milwaukee, WI), and an Mx8000 (Philips Medical System, Andover, MA). Scanning was performed from the thoracic inlet to the lung base, with the patient at full inspiration; intravenous contrast material was not used. Images were obtained using a level of −600 Hounsfield units (HU) and a width of 1500 HU (lung window). All high resolution CT images were reconstructed into 0.625-mm-thick sections, with a tube voltage of 120–140 kV, tube current of 200–400 mA, and a 512 × 512 matrix, using a bone algorithm axial reconstruction and filtered back projection algorithm. CT images used to diagnose GGN were re-reviewed by two thoracic radiologists (QG W, LF Z), each having more than 12 years of experience in reading chest CT images, blinded to the lesion pathology.

### Three-dimensional (3D) measurement of parameters

**Fig. 1:**
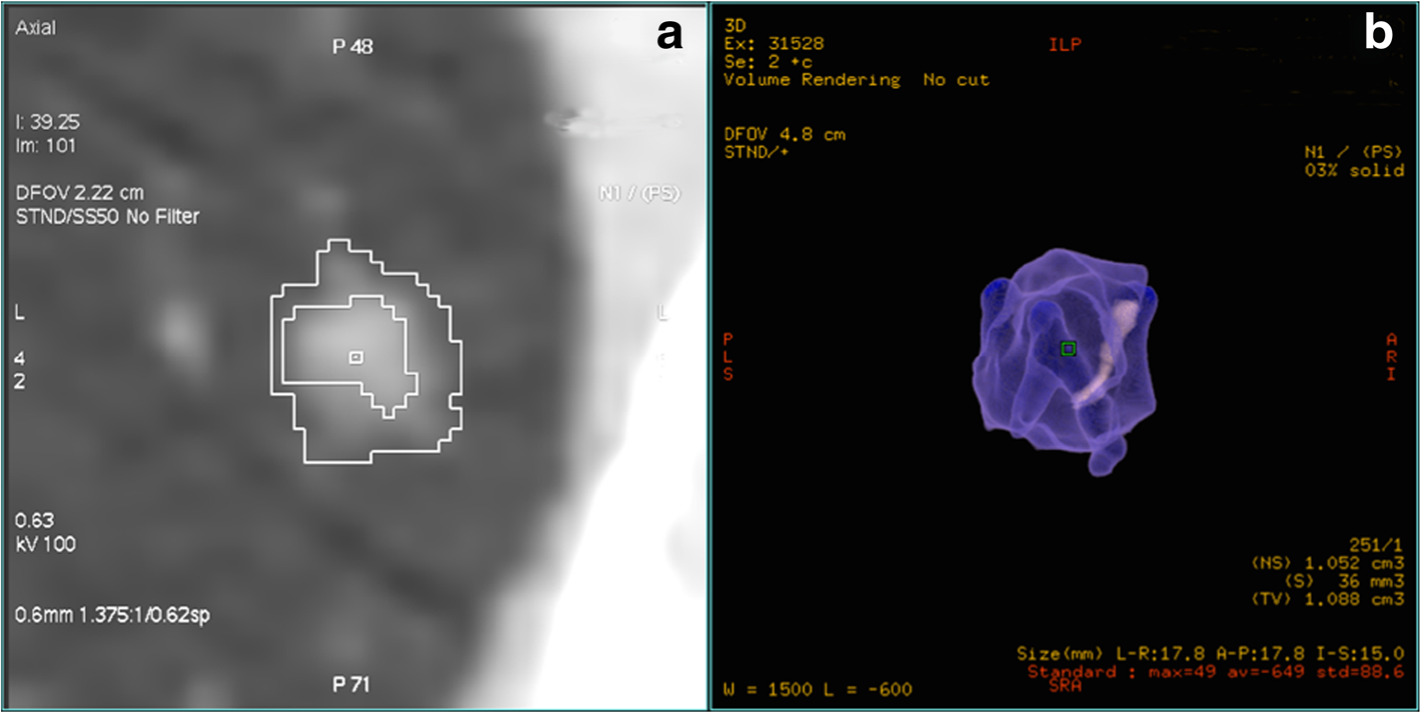
Segmentation and reconstruction of three-dimensional models of ground-glass opacity nodules. **a** Applied software automatically segmented nodule from surrounding normal lung tissue, marked by a *white tortuous line*. **b** Three-dimensional model of the nodule was measured, using pseudo-color, for the relevant parameters listed in the *bottom right corner*

The parameters used in our study, including GGN diameter, total volume (TV), PEAK and MAX, were automatically measured in a 3D reconstructive model using lung analysis software (Lung VCAR; GE Healthcare; USA) on a commercially available workstation (Advantage Workstation 4.3; GE Healthcare), as previously described [6–10]. Specific instructions and relevant documents could be found at www.gehealthcare.com/aw/applications/thoracic-vcar. This software can segment pulmonary nodules with ground-glass attenuation, and can automatically identify GGNs, in the X-, Y-, and Z-axis directions, from the surrounding normal lung tissue. The elimination of normal structures within or around the nodule, such as vessels or bronchioles, was performed using several image-processing techniques [11]. Diameters, TVs, and MAXs were automatically computed after the operator placed a marker on each nodule (Fig. 1). PEAKs were measured using CT number histograms produced by the advanced control window.

The M-P and V(M-P) values for all nodules were calculated. As the proliferation curve of cancer cells presents with a sigmoidal shape, the velocity of CT value changes are alterable during lung cancer progression. Therefore, the defined parameters only indicated the mean change in GGN CT velocity values throughout the three months of preoperative follow-up. The M-P and V(M-P) values were calculated according to the following equations:

1 $$ \mathrm{M}-\mathrm{P}=\mathrm{MAX}-\mathrm{P}\mathrm{EAK} $$

2 $$ \mathrm{V}\left(\mathrm{M}-\mathrm{P}\right)=\left(\left({\mathrm{MAX}}_2-{\mathrm{PEAK}}_2\right)-\left({\mathrm{MAX}}_1-{\mathrm{PEAK}}_1\right)\right)/\varDelta \mathrm{t} $$

**Fig. 2:**
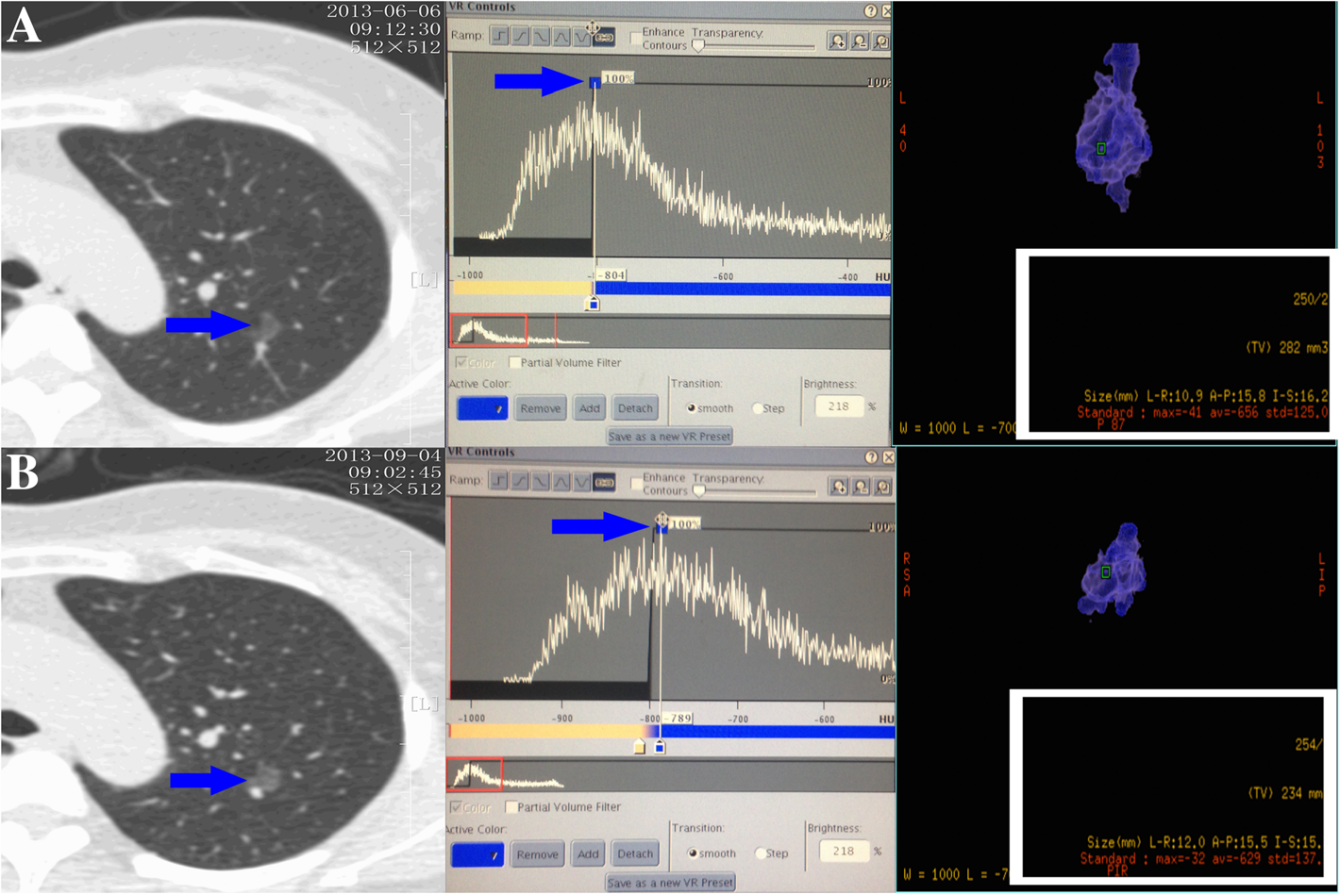
Example of a nodule visualized using computed tomography (CT), CT number histogram and three-dimensional model. **a** A ground-glass nodule (GGN) located in the upper lobe of left lung (*blue arrow*). The peak (*blue arrow*) CT number in the histogram was −804 HU and the maximum CT number measured automatically in the three-dimensional reconstructive model was −41 HU (*white box*). **b** The same GGN redetected after a 3-month follow-up (*blue arrow*). The peak (blue arrow) CT number had increased to −789 HU and the maximum CT number had increased to −32 HU (*white box*)

MAX_2_ and PEAK_2_ were measured after the follow-up visit, whereas MAX_1_ and PEAK_1_ were determined prior to the follow-up interval. The ∆t value in the above equation reflects the interval (in days) between the two CT scans (Fig. 2).

### Statistical evaluation

To assess variability, we calculated the 95% confidence interval (CI) for the limits of agreement using the Bland-Altman analyses. Gender, GGN subtype, GGN number and positions were analyzed using Chi-square tests. One-way ANOVA analysis was performed in terms of mean ages of the patients with GGNs. We compared both intra- and inter-observer variabilities; both showed normal distributions, according to a Kolmogorov-Smirnov test. As the variables were automatically measured by the software, the data variability reflectets software operation, such as marker placement on nodules rather than manual measurements. We calculated the mean coefficient of variation (CV) across all GGNs, with the CV being calculated as the standard deviation divided by the mean. Wilcoxon tests were performed, as appropriate for data with non-normal distributions, to compare the M-P and V(M-P) differences between PIAs and IACs. We also compared the M-P and V(M-P) values between pure and mixed GGNs as well as between solitary and multiple GGNs. Receiver operating characteristic curve (ROC) analyses were performed to assess the diagnostic specificities and sensitivities associated with the M-P and V(M-P) parameters. All statistical analyses were performed using SPSS, version 20.0 (IBM, Armonk, NY); *p-*values < 0.05 were considered statistically significant.

## Results

We retrospectively reviewed the medical data for 102 patients, including those with PIAs (AAH, n = 19; AIS, n = 24) and IACs (n = 59; including MIA, n = 37) (Table 1).

**Table 1:** Clinical and pathologic characteristics of all ground-glass opacity (GGO) nodules with different pathologic categories (*n* = 102)

Variables	PIA (*n* = 43)	AC (*n* = 59)	*P*
Gender (*n*)			0.063
Male	29	24	
Female	14	35	
Mean age (year)	53.14 ± 5.80	60.61 ± 10.68	0.001*
GGO subtype			0.226
Pure GGO	18	24	
Mixed GGO	25	35	
GGO number (*n*)			0.001*
Solitary	42	24	
Multiple	1	35	
Position			0.692
RUL	18	14	
RML	6	12	
RLL	0	14	
LUL	18	9	
LLL	1	10	

*P < 0.05

Classification according to the new IASLC/ATS/ERS International Multidisciplinary Lung Adenocarcinoma Classification system [31]: preinvasive adenocarcinoma (PIA) including atypical adenomatous hyperplasia (AAH) and adenocarcinoma in situ (AIS), adenocarcinoma (AC) including minimally invasive adenocarcinoma (MIA) and invasive adenocarcinoma (IAC)

RUL stands for Right Upper Lobe, RML stands for Right Middle Lobe, RLL stands for Right Low Lobe, LUL stands for Left Upper Lobe, LLL stands for Left Low Lobe

Gender, GGO subtype, GGO number and position were analyzed by Chi-square test

P indicates the p values for one-way ANOVA analysis in terms of the mean ages of all GGO nodules

### Measurement variability

**Fig. 3:**
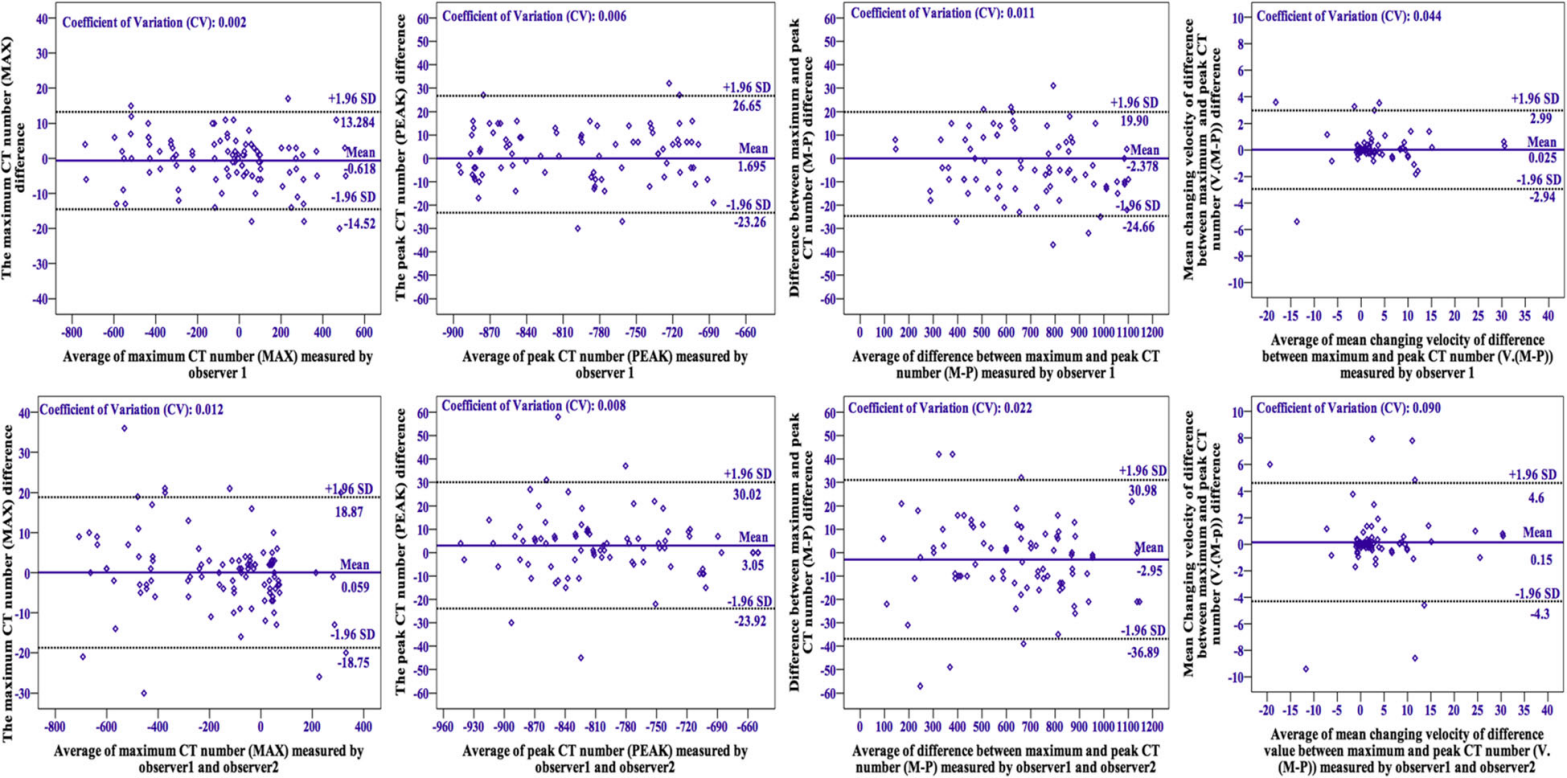
Bland-Altman scatterplots. The intra- (*upper*) and inter-observer (*lower*) variabilities are shown for the maximum CT number (MAX), peak CT number (PEAK), difference between maximum and peak CT numbers (M-P), and mean changing velocity of M-P (V. (M-P)). SD = standard deviation. *Low* measurement coefficients of variation indicate good reproducibility and error control

The 95% confidence intervals (CIs) for the limits of agreement of the measured parameters are shown in Table 2 and Fig. 3.

**Table 2:** Limits of agreement for the measured parameters

Measurement	Inter-observer variability (95% CI)	Intra-observed variability (95% CI)
MAX	−14.52, 13.28	−18.75, 18.87
PEAK	−23.26, 26.65	−23.92, 30.02
M-P	−24.66, 19.90	−36.89, 30.98
V(M-P)	−2.94, 2.99	−4.3, 4.6

*CI* confidence interval, *MAX* maximum computed tomography attenuation number, *PEAK* peak computed tomography number *M-P* difference between MAX and PEAK, *V (M-P)*, average change in M-P velocity

For the MAX measurements, the mean intra-observer CV was 0.002, and the mean inter-observer CV was 0.012. The mean intra- and inter-observer CVs for PEAK were 0.006 and 0.008, respectively, but were revised to 0.011 and 0.022, respectively, when converted to M-P. For the V(M-P) measurements, the mean intra-observer CV was 0.044, and the mean inter-observer CV was 0.090.

### M-P differences

**Fig. 4:**
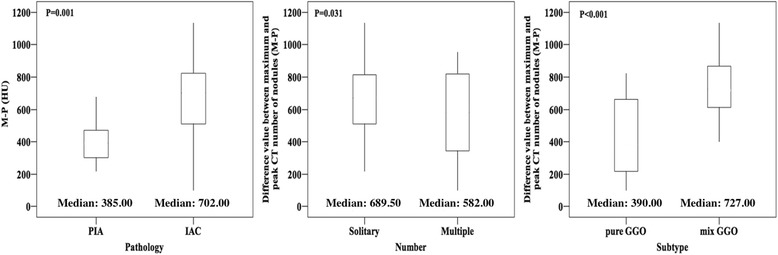
*Boxplots* comparing the differences between maximum and peak CT numbers (M-P) of ground-glass nodules (GGN) for distinguishing between pre-invasive adenocarcinoma (PIA) and invasive adenocarcinoma (IAC), between solitary GGNs and multiple GGNs, and between pure GGNs and mix GGNs. IACs, isolated nodules and mix GGNs had a significantly higher M-P values than PIAs, multiple nodules and pure GGNs respectively

The mean M-P value for GGNs with PIA (mean, 408.67 HU; range, 218.00–677.00 HU) was significantly smaller (*p* = 0.001) than that for GGNs with IAC (mean, 667.43 HU; range, 98.00–1136.00 HU). Additionally, we compared the M-P values in GGN nodules having different subtypes and numbers. A remarkably higher M-P (*p* = 0.031) difference was found for solitary GGNs (mean, 674.42 HU; range, 153.00–1136.00 HU) than for multiple GGNs (mean, 551.80 HU; range, 98.00–953.00 HU). Similarly, the M-P values for mixed GGNs (mean, 736.43 HU; range, 463.00–1136.00 HU) were significantly larger (*p* < 0.001) than those for pure GGNs (mean, 418.36 HU; range, 98.00–823.00 HU) (Fig. 4).

### V(M-P)

**Fig. 5:**
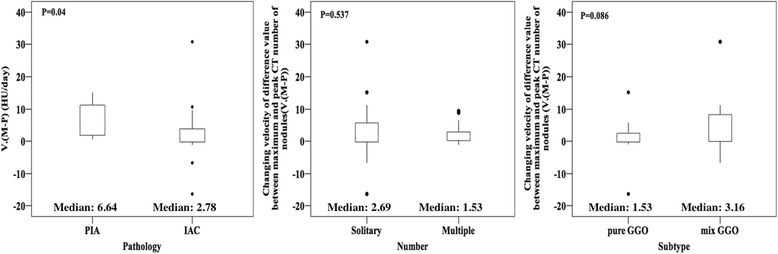
*Boxplots* comparing mean changes in the velocity of the difference between maximum and peak CT numbers (M-P) of ground-glass nodules (GGNs) for distinguishing pre-invasive adenocarcinomas (PIAs) from invasive adenocarcinomas (IACs), solitary GGNs from multiple GGNs and pure GGNs from mix GGNs. PIAs showed a remarkably higher V. (M-P) than did IACs. Significant differences did existed between solitary and multiple GGNs, as well as between pure and mix GGNs

The V(M-P) values for GGNs with PIA (mean, 6.92 HU/day; range, 1.00–15.00 HU/day) were significantly higher (*p* = 0.04) than for GGNs with IAC (mean, 2.36 HU/day; range, −16.4–30.74 HU/day). However, there was no significant difference in the values between solitary, and multiple nodules (p = 0.537) or between pure and mixed GGNs (p = 0.086) (Fig. 5).

### ROC analysis of M-P and V(M-P) for differentiating between PIA and IAC

**Fig. 6:**
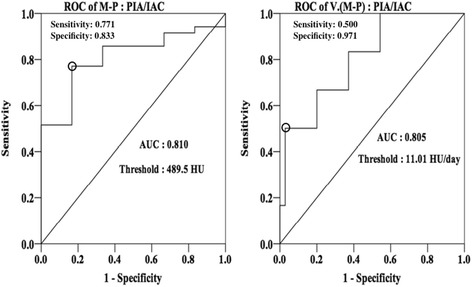
Receiver operating characteristic curves (ROC) of the differences between the maximum and peak CT numbers (M-P) and the mean changes in velocity of the M-P (V. (M-P)) of ground-glass nodules (GGNs) for differentiating between pre-invasive adenocarcinomas (PIAs) and invasive adenocarcinomas (IACs). Areas under the curves (AUC) for M-P and V. (M-P) were 0.810 and 0.805 respectively, including a relatively good sensitivity and specificity for differentiating the threshold values of 489.6 Hounsfield units (HU) and 11.01 HU/day respectively

**Fig. 7:**
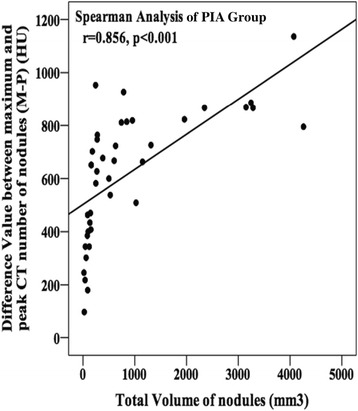
Correlation between the total volumes (TVs) of ground-glass nodules (GGNs) in the PIA group and the difference between the maximum and the peak CT numbers (M-P) of nodules using Spearman correlation analysis. Along with TV increasing, M-P values also increased, reflecting that the heterogeneity of the nodules, between the solid core and the principal, increased as well

The ROC evaluation showed areas under the curve (AUCs) for the M-P (0.810) and V(M-P) (0.805) values. The optimal cut-off points were defined as those closest to the upper left-hand corner of the curves [12], yielding thresholds of 489.5 HU and 11.01 HU/day for the M-P and V(M-P) parameters, respectively (Fig. 6). The sensitivities and specificities for the M-P values were 0.771 and 0.883 respectively, and those for the V(M-P) values were 0.500 and 0.971 respectively. Additionally, the Spearman correlation analysis for datasets with non-normal distributions, showed a significant (*p* = 0.001) positive correlation (0.856) between the M-P parameter and the GGN TV in the PIA group (Fig. 7).

## Discussion

As precursors of lung adenocarcinoma, GGNs undergo progression, similar to AAHs, AISs, MIAs, and IACs. Without invasion of the lung interstitium and lymph nodes, limited resections, such as wedge resections and segmentectomies, are appropriate for PIAs, including AAHs and AISs. For MIAs and IACs, some cases have shown invasion of the local lymph nodes, making standard lobectomies necessary [13–16]. Detecting potentially invasive malignant changes in GGNs can be challenging. To our knowledge, our use of an analysis of the M-P and V(M-P) parameters to determine GGN substructural features and changes to differentiate pre-invasive lesions from invasive ones is unprecedented. Traditionally, such differentiations have relied on VDTs or the average CT numbers for whole GGNs.

### Measurement variability

In this study, Bland-Altman analyses were used to evaluate potentially malignant GGNs. Measurements of the maximum and peak CT numbers using automatic 3D-reconstruction post-processing software resulted in low inter- and intra-observer CVs. The low variability resulted in a significantly improved ability to detect interior growth using CT numbers in different areas of the nodule, relative to overall descriptions of the diameter or volume of a subgroup of malignant GGNs. Because of the slow growth typical of GGNs, volume or density changes can be subtle, emphasizing the need for a precise measurement method. For solid nodules, Revel et al. concluded that two-dimensional measurements are unreliable [17]. As has been previously demonstrated for solid nodules, 3D volume measurements have lower intra- and inter-observer variabilities than do two-dimensional diameter measurements [18].

As described in Fig. 3, the intra-observer CVs of the MAX and PEAK values were as small as 0.002 and 0.006, respectively; the CVs increased to 0.011 and 0.044, respectively, for M-P and V(M-P) values. The relatively higher CV for M-P may have resulted from a cumulative or aggregative effect of variations in both the MAX and PEAK values. Furthermore, the CV for V(M-P) accumulated the variations in MAX, PEAK, and M-P. Secondly, all traditional CT parameters were measured using the same tube voltages, tube currents, and section thicknesses. Therefore, the algorithm in which the density of the same type of tissue was measured on the CT screen did not change significantly. Our proposed parameters were post-processing data that were subjectively measured by radiologists. Most importantly, as the parameters created in this study were new and not proficiently or regularly measured by our radiologists during clinical work, further research investigating more precise measuring methods and strategies needs to be completed.

### Difference between maximum and peak CT number (M-P)

We realized that GGNs are not homogeneous lesions, based on CT numbers, regardless of their subjectively judged subtype purity. In the presented study, the peak (PEAK) and maximum CT (MAX) numbers for nodules were combined to investigate the sub-structural densities of GGNs in our study. PEAK refers to the CT number associated with the greatest number of pixels in a nodule’s CT number histogram; these values are most likely exhibited in the main body of the GGN. Similarly, MAX refers to the CT numbers with the highest density and that are most likely located in the core, or solid part, of the GGN. Thus, the M-P values for mixed GGNs (mean, 736.43 ± 182.92 HU) were significantly larger (*p* < 0.001) than those for pure GGNs (mean, 418.36 ± 229.22 HU). Therefore, M-P values reflect the degree of homogeneity between the core and periphery of a GGN. Additionally, as illustrated in Fig. 7, when the TV of a GGN increases, the density of the principal structure more closely approximates that of normal lung parenchyma. This results in an increasing density difference between the solid core and the main body of the nodule increasing as well.

In the present study, the M-P values for PIAs (mean, 408.67 ± 150.23 HU) were significantly smaller (*p* = 0.001) than those for IACs (mean, 667.43 ± 243.40 HU). As suggested above, PIAs would be expected to be more homogeneous than IACs. During the progression of GGNs and lung adenocarcinoma, AAHs and AISs start proliferating so that their size and their ability to be distinguished from normal cells decrease. Using a Spearman correlation analysis, a positive coefficient of 0.856 (*p* < 0.001) was obtained between M-P values and the TV of GGN nodules. Therefore, PIA lesions tend to be smaller than nodules and exhibit density differences between the solid centers and the peripheral main bodies. IACs have larger volumes and are more invasive; they also have increased solid center densities and higher densities in the peripheral areas of the nodules. Additionally, an AUC of 0.810 simultaneously suggests both a favorable specificity and a favorable sensitivity, when an M-P value approaches the cut-off point of 489.5 HU, for distinguishing PIAs from IACs. If the preoperative M-P value for a GGN is larger than 489.5 HU, there is a greater possibility of the lesion being IAC. Otherwise, it could undergo further follow-up to achieve a more accurate assessment.

Lee et al. found that the average CT number of a GGN could be used to discriminate between an invasive lesion and a non-invasive one at −472 HU [19]. Similarly, Tamura et al. used the average CT number to evaluate GGN stability, suggesting that CT number cutoffs of −634.9 ± 15.3 HU and −712.1 ± 14.1 HU represented growing and stable GGNs, respectively [20]. Some authors have already investigated the average CT number for GGNs that allow pathologic differentiation. Although others have studied the histogram peak CT numbers of GGNs, they have shown great interest in peak patterns, 5 to 95th percentile CT numbers, skewedness, and kurtosis [21–30]; furthermore, all of these studies focused on the CT number for the whole GGN.

### Average V(M-P)

Follow-up is an important approach for assessing the pathological quality of GGNs. For example, VDT has been studied as an effective indicator for diagnosing pulmonary nodules. Oda’s research suggested that VDTs of 859.2 ± 428.9, 421.2 ± 228.4, and 202.1 ± 84.3 day were diagnostic of AAH, bronchioloalveolar carcinoma (BAC), and IAC, respectively [8]. Nevertheless, during a follow-up interval, the volume of most GGN lesions, especially for malignant ones, increases while the density also changes. If the VDT is used to evaluate the progression of the GGN exterior during a follow-up interval, then the use of V(M-P) should achieve a similar goal for the interior of the lesion. In the present study, the V(M-P) values of PIA lesions (6.92 ± 5.86 HU/day) were significantly larger (*p* = 0.04) than those for IAC lesions (2.36 ± 6.86 HU/day). The absence of a negative sign before the results suggests increasing heterogeneity.

During the follow-up of PIA lesions, the proliferation of pre-invasive carcinoma cells is restricted to small lesions that do not extend outwards. AAH was defined as a lesion with a well-defined boundary, produced by the proliferation of mildly to moderately atypical type II pneumocytes or Clara cells lining the alveolar walls and respiratory bronchioles. Gaps are usually seen between the cells, which consist of rounded, cuboidal, low columnar or “peg” cells with round to oval nuclei. For AIS, localized small adenocarcinomas with growth restricted to neoplastic cells along preexisting alveolar structures (lepidic growth), there is a lack of stromal, vascular, or pleural invasion [31]. Therefore, the interstitium is normal, without disordered structures or invasive malignant cells. Furthermore, the lung parenchyma, like the alveolar epithelium, continues to proliferate during the follow-up interval. Since only the small cells were growing rapidly, the density of the solid core area, on the CT screen, increased to higher values than did the main pixels associated with the nodule. Larger changes in the differences between the center and peripheral CT numbers were found in PIAs during the follow-up.

Malignant cells in IACs proliferated from multiple points throughout the nodule, invading the interstitium and filling it with mucous and isolated tumor cells so that, over time, the main part of the GGN showed an increasing density rather than only a solid core. Consequently, the V(M-P) values for IACs are remarkably smaller than those for PIAs, over the duration of the follow-up interval. An AUC of 0.805 was obtained, using the ROC assessment, for V(M-P) values allowing a differential diagnosis between PIAs and IACs, with a threshold of 11.01 HU/day. Accordingly, during the follow-up, if a GGN V(M-P) value larger than 11.01 HU/day is observed, PIA would be strongly suggested, rather than IAC.

### Study Strengths and Limitations

All measurements used in our study were obtained automatically using 3D procedure software on a commercially available workstation. Quantitative 3D measurements of small pulmonary nodules, on CT images, are attracting increased attention. Such models can accurately measure parameters, such as the mean diameter and volume as well as all of the variables associated with CT numbers, and can present the information in an intuitive manner. Automatic segmentation software may minimize observer differences and also shorten the evaluation times; and new computer algorithms that are better suited for this task have also been developed. Most major CT vendors have included a GGN segmentation option in their pulmonary evaluation software.

Additionally, previous researches referring to GGN substructure was confined to simple classifications of subjectively assessed pure and mixed subtypes. The present study focused on the internal quantitative features of GGNs by comparing differences between the densities of the solid cores and the main bodies, as well as their changes during the follow-up. This was fortunate, as it proved effective for differentiating the types. Given GGN homogeneity, internal density differences provide information regarding oncological behavior, in addition to VDTs.

We compared the diagnostic capabilities of M-P and V(M-P) values with PEAK and V-PEAK reported by ourselves previously [32]. We found that the AUCs in the ROC analysis of different parameters are very close to each other, notwithstanding a slightly higher value of PEAK. Nevertheless, after we concluded the several advantages and disadvantages of these two parameters, we believed that the M-P and V(M-P) characteristics, described in this present study, are superior to the PEAK and V-PEAK published at BJR. Our reasons are listed as follows: 1). According to other researches, such as Yasuhisa Ohde’s, the proportion of consolidation to GGO on high resolution CTs, at the respective maximum dimensions, was the best predictor of non-invasive peripheral lung adenocarcinoma [33]. Therefore, M-P takes the solid part of GGN into consideration to evaluate pathological properties of nodules, given that M-P indicates the differences between the CT numbers for the solid parts and of peripheral parts in most cases. However, evaluation by only PEAK, involved in previous study published at BJR, just represents and descripts either peripheral ground-glass part (when proportion of consolidation less than 50%) or solid part (when proportion of consolidation more than 50%), omitting part radiological information of GGN during diagnosis and evaluation. 2). All of previous studies involving radiological diagnoses of GGN treated it as a whole entity. Unprecedentedly, in the present study, we created these two particular indexes, M-P and V(M-P), from a new perspective of substructure of GGNs to evaluate its radiological and pathological properties. 3). With regarding to mix GGN, there must be two peaks in CT histogram when we measure PEAK. However, particularly, when consolidation of it equals to 50%, these two peaks would be the same height but different PEAK value, which makes it difficult and inappropriate to use PEAK only. 4). Additionally, PEAK would dramatically change when solid part of GGN near to 50% or so, affecting comparison between different individuals and different values of itself during follow-up. 5). Speaking of comparison between individuals, the same PEAK could be either CT number of solid part in mix GGN or peripheral ground-glass part in pure GGN but neglect different radiological and pathological properties.

Our study had several limitations. First, the sample size was relatively small as some of the patients with GGN nodules were followed-up outside of our hospital or only came to our hospital for surgical therapy. Second, considering that benign lesions changed greatly or disappeared from CT scans after follow-up, we only collected information on malignant nodules during this study. Third, the V(M-P) values, obtained using an equation similar to that used for calculating arithmetic means, only captured the average growth velocity of M-P values over the follow-up period. However, the rate of change for GGN features varies constantly. Lastly, as for Fig. 7, we just only analyzed the PIA group corresponding to ROC analysis because the relativity between total volume of invasive adenocarcinoma and its density is not such significantly high, considering its density in CT screen would not change so sensitive after advancing into invasive adenocarcinoma.

## Conclusions

In conclusion, from the perspective of GGN substructures, we found that differences between the MAX and PEAK CT numbers (M-P), respectively representing the densities of the solid core and main body part, and their changing velocities (V(M-P)) can effectively distinguish IAC from PIA.

## Abbreviations

3D: Three-dimensional
AAH: Atypical adenomatous hyperplasia
AIS: Adenocarcinoma in situ
AUC: Area under the curve
CI: Confidence interval
GGN: Ground-glass nodule
HU: Hounsfield units
IAC: Nvasive adenocarcinoma
MAX: The GGN maximum CT number
MIA: Minimally invasive adenocarcinoma
M-P: The difference between the maximum and peak CT numbers of nodules
PEAK: GGN peak CT number
PIA: Pre-invasive adenocarcinoma
ROC: Receiver operating characteristic
TV: Total volume
VDT: Volume-doubling time
V(M-P): Mean velocity of changes in the M-P values
